# AI versus human-designed exam questions in undergraduate dental education: A randomized controlled study

**DOI:** 10.6026/973206300214701

**Published:** 2025-12-15

**Authors:** Preeti Mankar, Deepti Vijay Mankar, Saara Khiyani, Manish Kumar, Varunraj Jadhav, Saloni P Shah

**Affiliations:** 1 Department of Prosthodontics, Crown and Bridge & Implantology, Nanded Rural Dental College & Research Centre, Nanded, Maharashtra, India; 2 Department of Pathology, American University School of Medicine, Aruba; 3 Department of Conservative Dentistry and Endodontics, Guru Gobind Singh Dental College, Burhanpur, India; 4 Department of Prosthodontics, Teerthanker Mahaveer Dental College and Research Centre, Bagadpur, Uttar Pradesh, India; 5 Department of Prosthodontics and Crown & Bridge,Yogita Dental College and Hospital, (Khed), Ratnagiri, India; 6 Department of Prosthodontics, Esic Dental College and Hospital, Kalaburagi, India

**Keywords:** AI-generated questions, assessment, dental education, exam questions, human-designed questions

## Abstract

The comparison of the effectiveness and fairness between AI-generated and human-designed exam questions in the context of undergraduate dental education is relevant. Therefore, it is of interest to examine the use of AI-generated versus human-designed exam questions in undergraduate dental education. A randomized controlled trial involving 289 students was conducted to compare the effectiveness and fairness of both formats. Results showed no significant difference in overall student performance between the two groups. However, students preferred human-designed questions for their clarity and familiarity. Thus, the role of AI-generated exam questions in undergraduate dental education compared to traditional human-designed questions remains a challenge.

## Background:

In recent years, the integration of artificial intelligence (AI) into education has sparked significant interest, particularly in the context of assessment and examination [1]. The potential of AI to enhance the educational experience has led to its increasing use in various fields, including medical and dental education [2]. As dental education continues to evolve, it is crucial to explore new ways of designing and implementing effective assessment tools. Traditionally, exam questions in dental education have been developed by educators, who use their expertise and experience to create assessments that align with the curriculum and test students' knowledge and skills. However, the advent of AI-generated exam questions presents an intriguing alternative [3]. AI-based systems can generate exam questions by analyzing large sets of data, identifying patterns in student performance and understanding the key topics and concepts that are most important for students to learn. This has the potential to provide a more personalized, data-driven approach to assessment, which could lead to more accurate and efficient evaluations of students' abilities [4]. AI can also help in the creation of questions that are varied in format, such as multiple-choice questions, short-answer questions, and case-based questions, allowing for a broader range of skills to be assessed. Moreover, the ability to continuously adapt and improve question quality based on real-time data could provide significant advantages in ensuring that assessments remain relevant and effective [5]. However, despite the promise of AI in the development of exam questions, there are concerns regarding the validity and reliability of AI-generated assessments. The main challenge lies in determining whether AI can design questions that are as accurate, fair, and comprehensive as those created by human educators [6]. While AI has the capacity to analyze large amounts of data and generate questions based on patterns, it may lack the nuanced understanding that a human educator brings to the table. Educators often have a deep understanding of how students think and the common misconceptions they may hold, which can inform the design of well-targeted questions [7]. Additionally, human educators have the ability to design questions that consider not only the cognitive level of the content but also the pedagogical goals of the course, something that may be difficult for an AI system to replicate fully [8]. Therefore, it is of interest to determine whether AI can effectively replace or complement traditional question design in dental education and to assess its potential to enhance the quality and fairness of student assessments.

## Methodology:

This study was conducted at the Nanded Rural Dental College and Research Centre, Nanded, which provides an ideal setting for examining the use of AI-generated versus human-designed exam questions in undergraduate dental education. The study employed a randomized controlled design to assess the effectiveness, fairness, and educational impact of AI-based and human-designed exam questions. The participants in this study were undergraduate dental students enrolled at Nanded Rural Dental College and Research Centre. A total sample size of 289 students of 2nd, 3rd, 4th year BDS were targeted based on the available cohort size for the academic year 2023-2024. The students were randomly assigned to two groups: one group that received exams with AI-generated questions and another that received exams with traditionally human-designed questions. This randomization helped ensure that any differences in student performance could be attributed to the type of exam questions rather than individual characteristics or biases. The AI technology used in this study for manuscript preparation was based on OpenAI's GPT-4 model. GPT-4 is a highly advanced natural language processing tool designed to assist in generating, analyzing, and editing written text, offering a high degree of linguistic understanding and contextual coherence. The exam questions for both groups were designed to be equivalent in terms of content, difficulty, and coverage of the curriculum. The AI-generated exam questions were created using a sophisticated artificial intelligence system capable of analyzing student performance data, identifying key concepts from the syllabus, and generating a variety of question types (e.g., multiple-choice, short answer, case-based). On the other hand, the human-designed exam questions were crafted by experienced faculty members at the dental college, who used their expertise and knowledge of common student misconceptions to design comprehensive and accurate questions. In addition to the exam performance data, feedback from students was gathered using a Likert scale, which included questions on the clarity of exam questions, overall satisfaction, and their perceived difficulty of the exams. The scale ranged from 'Strongly Agree' to 'Strongly Disagree,' allowing for a structured analysis of student perceptions. Both sets of exam questions were tailored to assess a wide range of dental knowledge, including theoretical understanding, clinical reasoning, and problem-solving abilities. The exams for both groups were structured to maintain similar formats and included a comparable number of questions to ensure fairness in evaluation. Student performance on both sets of exams was recorded and analyzed to compare outcomes between the AI-generated and human-designed exam groups. Key performance indicators such as the overall exam scores, question-specific scores, and the time taken to complete the exams were collected for analysis. In addition to the quantitative data, student feedback was also gathered to assess their perceptions of the two types of exam questions. This feedback was collected through a structured questionnaire that included both closed and open-ended questions regarding students' experiences with the exams, the clarity and difficulty of the questions, and their overall satisfaction with the assessment format. The data from the student performance, as well as the feedback, were analyzed using appropriate statistical methods. Descriptive statistics were used to summarize student scores and demographic information. Inferential statistics, including t-tests and chi-square tests, were employed to compare the performance of the two groups and to assess the significance of any differences observed. The feedback data were analyzed qualitatively to identify themes and patterns in student perceptions regarding the two types of exam questions. This study was reviewed and approved by the Institutional Ethics Committee of Nanded Rural Dental College and Research Centre. Written informed consent was obtained from all participants prior to their participation in the study. By comparing the effectiveness and student perceptions of AI-generated versus human-designed exam questions, this study aims to provide valuable insights into how AI can be integrated into the assessment process in dental education.

## Results:

The analysis of the data from the study comparing AI-generated and human-designed exam questions yielded insightful findings in terms of student performance, feedback, and overall assessment outcomes. The results were based on the scores of 289 undergraduate dental students, who were randomly assigned to either the AI-generated or human-designed question groups. The following tables summarize the findings related to student performance, the time taken for exam completion, and the feedback collected from students. The first set of results focuses on the overall performance of students in both groups. The data revealed that there was no significant difference in the mean scores between students who took the AI-generated exams and those who took the human-designed exams. This suggests that both types of exams were equally effective in evaluating the students' knowledge and understanding (Table 1 - see PDF). In order to understand if students performed differently on specific types of questions, a breakdown of scores by question type was analyzed. The results show that students in the AI-generated group scored slightly higher on multiple-choice questions, while those in the human-designed group performed better on case-based questions. This suggests that question format might play a role in how students engage with different types of exam content (Table 2 - see PDF). An important aspect of the study was the time taken by students to complete their respective exams. The data indicated that students taking the AI-generated exam took, on average, slightly longer to complete the questions, potentially due to the complexity and structure of the questions generated by the AI system. However, the difference in time was not substantial enough to indicate a significant impact on the exam process (Table 3 - see PDF). The feedback data, collected through a Likert scale, revealed that a higher percentage of students in the human-designed group reported clearer questions compared to the AI-generated group. Table 4 (see PDF) shows the breakdown of these responses, with students rating the clarity and difficulty of questions on a five-point scale (Table 4 - see PDF). Overall student satisfaction was assessed through a set of survey questions. The results show that, while the majority of students were satisfied with both types of exams, there were a higher percentage of students in the human-designed group who felt more confident in their understanding of the material. Additionally, students in the AI-generated group expressed more uncertainty about the relevance of some of the questions (Table 5 - see PDF).

There was no significant difference in the overall scores between the two groups (AI-generated and human-designed), suggesting that both exam formats were equally effective in assessing student knowledge. The AI-generated exam group performed better on multiple-choice questions, while the human-designed exam group had higher scores on case-based questions. Although the AI-generated exam took slightly longer to complete, this difference was not substantial enough to affect overall performance or cause significant delays. Student feedback indicated that human-designed questions were clearer, and students expressed more confidence in their understanding of the material when taking these exams. However, the majority of students in both groups were generally satisfied with the exam formats. In addition to the descriptive statistics, inferential statistical tests were applied to compare the performance of the two groups (AI-generated vs. human-designed questions). A t-test was conducted to compare the mean scores of both groups, and no significant difference was found between the AI-generated exam (M = 78.45, SD = 7.56) and the human-designed exam (M = 77.92, SD = 8.12). This suggests that both sets of questions were equally effective in assessing student knowledge and understanding. Further, a chi-square test was used to analyze the categorical data from the feedback regarding question clarity and overall satisfaction. The results indicated no significant differences in overall satisfaction between the two groups (χ^2^ = 0.456, p = 0.710). This suggests that the students' perceptions of satisfaction with the exam format were similar across both groups, despite the slight preference for human-designed questions in terms of clarity. The results from this study offer valuable insights into the potential benefits and limitations of using AI-generated exam questions in dental education. While AI systems can generate effective questions, the nuanced understanding and clarity offered by human-designed questions remain an important factor in student satisfaction and performance.

## Discussion:

The findings of this study align with, and in some cases, diverge from previous research in the area of AI-based education assessments. Below, we compare the key results of this study with five previous studies examining the effectiveness of AI in educational assessments. The statistical analyses conducted using t-tests and chi-square tests showed no significant difference in overall exam performance between the AI-generated and human-designed groups. However, the feedback from students, gathered through a Likert scale, indicated that students preferred human-designed questions for their clarity and perceived fairness. Law *et al.* (2025) [9] conducted a study to evaluate the effectiveness of AI-generated exam questions in medical education, focusing on multiple-choice questions (MCQs). They found that AI-generated questions were as effective as those created by human educators in assessing basic medical knowledge. Similar to our findings, they reported that students performed equally well on both sets of questions. Our study extends this finding by exploring a wider range of question types, including short-answer and case-based questions, and confirming that while AI-generated MCQs performed well, students in the human-designed group outperformed AI-generated questions in more complex question formats such as case-based assessments. This suggests that AI might struggle to capture the nuanced clinical reasoning tested in case-based questions, which aligns with our findings of slightly better performance in the human-designed group in that category. Ma *et al.* (2025) [10] explored the use of AI for automatic generation of dental examination questions. They found that while AI could create accurate and varied questions, students reported difficulty in understanding some of the questions, particularly those involving clinical scenarios. This observation mirrors the feedback we received, where students in the AI-generated group reported challenges with the clarity of some questions, especially those involving more complex dental cases. While our results showed no significant difference in overall performance, the clarity of questions remains a crucial factor, as suggested by Dandekar and Joshi's study. Their work emphasizes the need for continuous improvement in AI algorithms to ensure clarity and relevance, a challenge that AI still faces in comparison to human-designed questions. Williams (2024) [11] *et al.* focused on the comparison between AI and human-designed assessments in higher education, particularly in undergraduate programs. Their findings were consistent with ours in terms of overall student performance, where no significant differences were observed. However, they also reported that AI-generated assessments took slightly longer for students to complete due to the nature of question complexity. Our study's results, showing that students taking the AI-generated exam took more time, align with Liu *et al.*'s conclusion. This may suggest that AI-generated questions, while effective, could be more time-consuming due to their complexity or the need for students to interpret them more carefully. Esmaeilzadeh *et al.* (2021) [12] conducted a study that examined the effectiveness of AI in designing assessments for medical students. Their study found that students performed similarly in both AI-generated and human-designed exams, with no significant advantage to either format in terms of final scores. However, they did note that the satisfaction levels were higher for human-designed questions, particularly regarding their perceived fairness and clarity.

This finding echoes our results, where students in the human-designed group expressed greater satisfaction with the clarity of questions. These results suggest that while AI is capable of generating effective questions, human-designed questions are still preferred by students for their clarity and alignment with their learning experiences. Baxmann *et al.* (2025) [13] explored the integration of AI in creating adaptive learning assessments in dentistry. They found that AI could create personalized assessments based on student performance, leading to improvements in long-term retention of knowledge. While the focus of their study was on adaptive assessments, our study's findings highlight an important point about the potential of AI to tailor exam questions to individual students. Our research did not specifically test adaptive learning or personalized questions, but the potential for AI to enhance customization in assessments remains a key avenue for future exploration. While the results suggest that AI-generated questions can serve as a valid alternative to human-designed questions in terms of academic outcomes, several important points must be considered. First, the clarity of AI-generated questions is still an area that requires improvement. While AI can generate complex questions, it may lack the ability to design questions that are as clearly articulated as those created by human educators who have a deeper understanding of how students learn and what common misconceptions they may have. Additionally, the satisfaction levels of students are influenced not only by question clarity but also by the perceived fairness of the assessment. Human-designed questions based on the educators' experiences and understanding of the course content, were preferred by students for their familiarity and clarity. This suggests that, for AI to be fully integrated into dental education assessments, it must evolve to create questions that are more in line with students' expectations and the nuances of educational content. Moreover, AI's potential to generate personalized or adaptive assessments, as suggested by Vardhe *et al.* (2025) [14], could be a future avenue to enhance its role in dental education. Adaptive AI assessments could allow for more precise evaluations based on individual learning progress, which may improve both short-term performance and long-term retention of knowledge.

## Conclusion:

AI-generated exam questions are comparable to human-designed questions in terms of student performance but may require improvements in clarity and student satisfaction. Human-designed questions currently offer more precise alignment with students' expectations and clarity. However, more data is to enhance AI's ability to create adaptive and clear assessments in dental education.
